# How Effective Are Non-Operative Intra-Articular Treatments for Bone Marrow Lesions in Knee Osteoarthritis in Adults? A Systematic Review of Controlled Clinical Trials

**DOI:** 10.3390/ph15121555

**Published:** 2022-12-14

**Authors:** Alexander C. Kleinschmidt, Ambrish Singh, Salman Hussain, Gregory A. Lovell, Anna Wong Shee

**Affiliations:** 1 Wakefield Sports + Exercise Medicine Clinic, Ground Floor, 120 Angas Street, Adelaide, SA 5000, Australia; 2 Menzies Institute for Medical Research, University of Tasmania, Hobart, TAS 7000, Australia; 3 Czech National Centre for Evidence-Based Healthcare and Knowledge Translation, Institute of Biostatistics and Analyses, Faculty of Medicine, Masaryk University, 62500 Brno, Czech Republic; 4 Research Institute for Sport and Exercise, University of Canberra, Bruce, ACT 2617, Australia; 5 Deakin Rural Health, Deakin University, Warrnambool, VIC 3280, Australia; 6 Grampians Health, Ballarat, VIC 3280, Australia

**Keywords:** knee osteoarthritis, sprifermin, autologous protein solution, systematic review

## Abstract

Knee osteoarthritis (KOA) is a progressive joint disease and a leading source of chronic pain and disability. OA-bone marrow lesions (BMLs) are a recognised aetiopathological feature of KOA. Several intra-articular injectable therapies are recommended and used for management of KOA. This systematic review assessed the efficacy and safety of intra-articular therapies for improving OA-BMLs and reducing pain in adults with KOA. The study was conducted following registered review protocol (PROSPERO CRD42020189461) and six bibliographic databases, and two clinical trial registries were searched. We included eight randomised clinical trials involving 1294 participants, reported in 12 publications from 2016 to 2021. Two studies of sprifermin, one of autologous protein solution (APS) and one of high-dose TissueGene-C, reported a positive effect on OA-BMLs under 1-year follow-up. Two studies with corticosteroids reported mixed findings with no beneficial effect beyond 14 weeks of follow-up. One study assessing platelet-rich plasma found no significant improvement in OA-BMLs at 12 months follow-up. Knee pain was improved in two studies evaluating TissueGene-C and one study assessing APS; the remaining studies found no improvement in knee pain. Overall, we found mixed evidence on the efficacy of intra-articular therapy for improving OA-BMLs in KOA. Additional studies with long-term follow-up are needed to confirm the effect of various intra-articular therapies on OA-BMLs in KOA.

## 1. Introduction

Osteoarthritis (OA) is a chronic, progressive, and painful condition that affects many joints, including the knee. Knee OA (KOA) is the most common form of OA and is characterised by an inflammatory-degenerative process of all joint structures, which involves reduced physical activity and social and occupational functioning, mainly due to pain, the primary symptom [1]. It is the leading cause of disability in OA patients worldwide and the predominant condition leading to total knee replacement (TKR) surgery from a global perspective [2]. 

Despite the substantial global burden of KOA, no disease-modifying osteoarthritis drugs (DMOADS) are available to treat OA [3]. The reasons are multifactorial and include multiple heterogeneous causes of OA, which are difficult to target given the multiple pathways of causality [3]. Furthermore, dry biomarkers, such as traditional radiography, are relatively insensitive to early KOA changes [3,4]. The initial treatment of KOA is conservative and includes targeted exercise programs, such as Good Life with osteoArthritis: Denmark (GLA:D^®^) [2,5], in conjunction with pharmacological interventions such as paracetamol and, in selective cases, nonsteroidal anti-inflammatory drugs (NSAIDs) [2]. With regard to conservative treatments, the use of non-pharmacological interventions such as knee rehabilitation exercises, including the combination of aerobic exercise, strengthening, neuromuscular training, isometric exercises, and pharmacological interventions such as intra-articular therapies (corticosteroids and hyaluronic acid) are commonly recommended by the guidelines. [2] For patients with chronic knee pain and functional loss, TKR is an effective option for many individuals. However, it is expensive and may be prevented or delayed if KOA is well managed conservatively.

KOA is a disease of the whole joint [6], and the role of subchondral bone in the pathogenesis of KOA has attracted increasing attention [7]. Bone marrow lesions (BMLs), referred to previously as bone marrow oedema, are recognised as important features of KOA [8]. However, BMLs are not exclusive to KOA and not all BMLs in patients with KOA are OA-BMLs [9]. OA-BMLs are those that are adjacent to articular cartilage and without any visible fracture line [9]. Magnetic resonance imaging (MRI) is the most sensitive modality for detecting OA-BMLs [4,10]. Several studies in patients with KOA have found knee pain to be positively associated with MRI-detected OA-BMLs. For example, Zhang et al. found that changes in OA-BMLs are associated with knee pain and that the decrease in OA-BML size is associated with a reduction in knee pain in patients with KOA [11]. Given that OA-BMLs occur early in subchondral bone and reverse earlier than cartilage degradation [12], treatments focused on targeting bone, shrinking OA-BMLs, and reducing focal contact stress across the joint can have a profound impact on reducing pain and progression to surgery [11].

Several interventions delivered through the intra-articular route are recommended by guidelines for the management of KOA [2]. Researchers have evaluated intra-articular treatments such as hyaluronic acid, glucocorticoids [13], autologous protein solution injections [14], platelet-rich plasma [15], human bone marrow mesenchymal stem cells [16], gene therapies [17], and human recombinant fibroblast growth factor (sprifermin) in patients with KOA [7]. Intra-articular therapies can be administered by direct injection into the knee in the clinic, for example, platelet-rich plasma [15], or by a surgical procedure involving a general anaesthetic such as bone marrow aspirate concentrate [16]. Surgical procedures are costly and more complex, with the potential for significant side effects and can only be used for certain cartilage defects [17]. Therapies administered in a clinical setting offer many patients a more convenient and accessible option.

Currently, intra-articular injectable therapies as treatments for KOA are in widespread use worldwide. The autologous blood product platelet-rich plasma (PRP), for example, produces high concentrations of growth factors and has been shown to have favourable pain and functional outcomes in KOA [15,18,19]. From a patient well-being perspective, since OA-BMLs are shown to be associated with pain and pain is one of the main reasons individuals progress to TKR, there is a need for a better understanding of how effective intra-articular injectable treatment options are for improving OA-BMLs and knee symptoms in patients with KOA. The purpose of this systematic review was to synthesise the evidence on the efficacy and safety of available non-surgical intra-articular injectable treatments. The research question for this review was “How effective are non-operative intra-articular injectable treatments for the improvement of OA-BMLs and reducing pain in adults with KOA?”.

## 2. Materials and Methods

This systematic review was performed following our a priori registered protocol on PROSPERO (registration number CRD42020189461) [20] and reported according to the Preferred Reporting Items for Systematic Review and Meta-Analyses (PRISMA) statement [21].

### 2.1. Criteria for Study Inclusion

Studies were included if they compared intra-articular injectable non-operative interventions with placebo or other active treatments for KOA in an adult population with at least one OA-BML on MRI. Eligible study designs included randomised, quasi-randomised, or non-randomised controlled clinical trials. Sub-group analysis and post hoc analysis were also eligible. No restrictions were placed on the type of publication (full-text papers or conference abstracts), provided they had reported measuring the required outcomes.

### 2.2. Criteria for Study Exclusion

We excluded studies with no comparison group and those published in a language other than English.

### 2.3. Population, Intervention, Comparator and Outcomes

A detailed account of the Population, Intervention, Comparator, and Outcomes (PICO) is provided in the published protocol [20]. Briefly, the population constituted adult (≥18 years) humans with KOA; the intervention group for this review was intra-articular injectable non-operative therapy, and the comparator group was a placebo or any active intra-articular pharmacological intervention.

The primary outcomes of interest included structural changes (maximal area/volume) of OA-BMLs, determined at baseline and treatment intervals measured by quantitative or semi-quantitative measurements from MRI. The secondary outcomes of interest included changes in knee pain intensity determined at baseline and at treatment intervals measured by validated patient-reported outcome measures (PROMS) such as the Knee Injury and Osteoarthritis Outcome Score (KOOS); Western Ontario and McMaster University Arthritis Index (WOMAC); and changes in health-related quality-of-life (HRQoL) were assessed using the Assessment of Quality of Life-8 Dimension score (AQoL-8D) and the 36-Item Short Form Health Survey score (SF-36).

### 2.4. Search Strategy and Study Selection

Systematic and comprehensive searches were conducted from database inception to 16 May 2022 in the bibliographic databases Ovid MEDLINE^®^, Embase, CENTRAL, CINAHL, SPORTDiscus and pEDro. Additionally, ClinicalTrials.gov and the Australian New Zealand Clinical Trials Registry (ANZCTR) were also searched. The search strategy was first optimised for MEDLINE and then adapted for other databases. The search strategy for MEDLINE is shown in Supplement Table S1. The complete search strategy, including MeSH terms, was developed and validated by the first author (A.K.) with assistance from a Medical Librarian. Bibliographic database searches were supplemented by hand-searching the reference lists of included articles and by contacting study authors.

Search results were imported into Covidence (Veritas Health Innovation, Melbourne, Australia), and two reviewers (A.K. and A.W.S.) screened the titles and abstracts of all articles against the eligibility criteria. Full-text copies of studies identified by the title/abstract screen as having met the inclusion criteria were obtained. Any disagreements or conflicting decisions were resolved through discussion and consensus with the other authors. Reasons for excluding studies were documented.

### 2.5. Data Extraction and Risk of Bias Assessment

Relevant data were extracted independently for each included study by two authors using a prespecified MS Excel-based data extraction template. The data extracted included publication details, study design, follow-up duration, population, intervention details, key outcomes, conclusion, etc.

Two reviewers (S.H. and A.S.) independently assessed the risk of bias in the included studies according to the Cochrane Handbook 5.0.1 RCT risk of bias assessment (RoB-I) tool [22]. As per the Cochrane RoB-I tool, the bias was assessed for items such as sequence generation, allocation concealment, blinding of participants, study personnel, outcome assessors, incomplete outcome data, selective outcome reporting, and other potential sources of bias.

### 2.6. Data Synthesis and Statistical Analysis

Due to the heterogeneity among included studies in terms of intervention, doses, duration of follow-up, and outcome reporting (OA-BMLs reported as a continuous outcome as well as categorical outcome), a meta-analysis was not deemed appropriate. Hence, we analysed the data qualitatively and presented it in the form of a narrative synthesis.

## 3. Results

**Figure 1:**
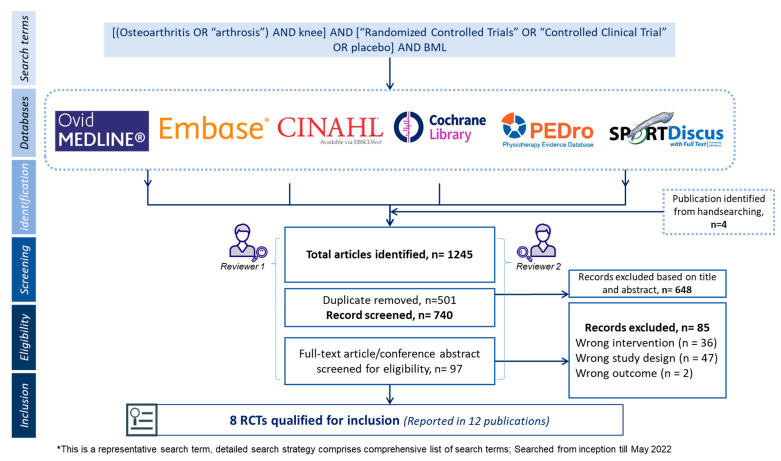
Flow chart describing method and inclusion of studies.

Following database searches, hand-searching references, and trial registry screening, 1245 records were identified (Figure 1). After removing duplicates, 740 records had titles and abstracts screened, and 97 full-text articles were screened for eligibility. After the full-text screening, eight studies reported in 12 publications met the eligibility criteria and were included in the review (Supplement Figure S1: PRISMA 2020 flow diagram).

### 3.1. Study Characteristics

The characteristics of included studies are summarised in Table 1. Of the eight included studies, two each used intra-articular injectable sprifermin [7,23,24,25], corticosteroids [13,26] and TissueGene-C [17,27,28,29], and one each used platelet-rich plasma [15] and APS [14]. All included studies were registered in clinical trial registries and were published between 2016 and 2021. Two studies were multicentre, multinational trials [7,25], two studies were from the US [26,29], and the remaining studies were conducted in Italy [14], South Korea [17], Australia [15], and Denmark [13]. The follow-up period ranged between 14 weeks to 24 months [7,13,26]; the largest trial consisted of 549 patients with KOA [7].

Four studies used the American College of Rheumatology (ACR) classification criteria for the diagnosis of KOA [7,13,25,26], and one study used the International Cartilage Repair Society (ICRS) criteria [17]. Additionally, seven studies [7,14,15,17,25,26,29] used Kellgren and Lawrence (KL) grade, and one study [13] reported using the Ahlback grading system to grade the severity of KOA and to classify the participants. The majority of the studies included participants with KL grade 2 to 3 KOA [7,14,15,25,26,29].

### 3.2. Risk of Bias

**Figure 2:**
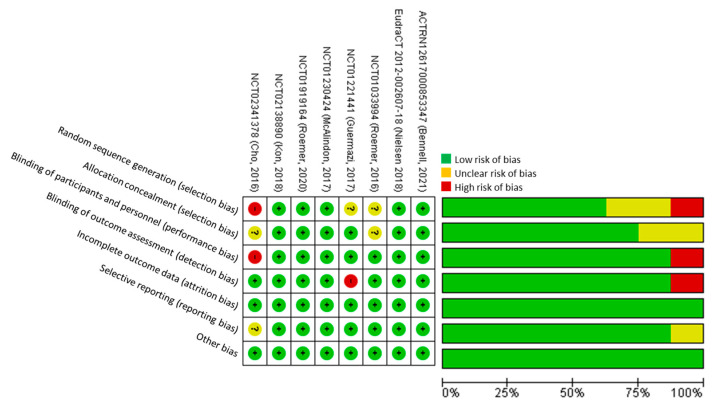
Cochrane risk of bias assessment.

The overall risk of bias in included studies was low, with five trials assessed as having a low risk of bias according to the Cochrane RoB-1 tool. Three studies were considered at risk of bias due to one or more RoB-1 tool domains (High risk: random sequence generation, blinding; Unclear risk: random sequence generation, selective reporting allocation concealment) being at high or unclear risk of bias (Figure 2).

### 3.3. Primary Outcome—Effect of Intra-Articular Treatments on MRI-Assessed OA-BMLs

In all included studies that used MRI to assess the outcomes of OA-BMLs, four of those studies assessed change in OA-BML size using a modified Whole-Organ Magnetic Resonance Imaging Score (WORMS) system [7,17,25,29], while one study used MRI Osteoarthritis Knee Score (MOAKS) to assess BMLs [14]. The remaining studies did not specify using any specific type of MRI scoring tool (Table 1 and Table 2). None of the studies assessed change in OA-BMLs as a primary outcome.

### 3.4. Sprifermin

Among the two studies that used intra-articular injectable sprifermin [7,25], Roemer et al. (2020) reported a positive effect of sprifermin in OA-BML on the patellofemoral joint (PFJ); however, there was no significant difference observed in OA-BML changes when accounted for the entire knee at 24 months of follow-up (*p* > 0.05) [7]. In an earlier study by Roemer et al. (2016), the OA-BMLs analysed for the whole knee showed significant (*p* = 0.042) improvement from 6 to 12 months but not from baseline to 6 months or 12 months (*p* = 0.237) [25].

### 3.5. Corticosteroids

Two studies evaluating corticosteroids [13,26] observed similar findings and reported no significant improvement in OA-BMLs. While Nielsen et al. observed a significant improvement in OA-BML volume at 14 weeks (mean difference: −3.8; 95% CI: −7, −0.5; *p* = 0.03), the difference in OA-BML volume levelled out at the 26 week follow-up (mean difference: −0.8; 95% CI: −4.4, 2.8; *p* = 0.65) [13]. Similarly, in a study evaluating triamcinolone, McAlindon et al. reported no significant improvement (*p* = 0.80) in OA-BMLs between the triamcinolone and saline groups at 24 months follow-up [26].

### 3.6. TissueGene-C

Two studies assessing TissueGene-C (TissueGene Inc., Rockville, MD), a 3:1 mixture of non-transduced allogeneic human chondrocytes and allogeneic human chondrocytes transduced to express transforming growth factor (TGF)-β1, reported mixed findings [17,29]. Cho et al. compared high-dose TissueGene-C with low-dose TissueGene-C and reported an improvement in OA-BMLs in the high-dose group (25% vs. 22%) at 12 months follow-up [17,28]. In contrast, Guermazi et al. found no significant difference (*p* = 0.237) in OA-BML progression between TissueGene-C and placebo at a similar follow-up duration [27,29].

### 3.7. Platelet-Rich Plasma and Autologous Protein Solution

Bennell et al. compared platelet-rich plasma against placebo in patients with symptomatic medial KOA (KL grade 2–3). The study found no significant (*p* = 0.31) benefit of platelet-rich plasma at 12 months follow-up for the reduction in OA-BML progression (24.3% vs. 18.9%) [15].

Kon et al. compared APS with placebo and reported a beneficial effect of APS on OA-BMLs in patients with KOA. At 12 months follow-up, the study found a significant (*p* = 0.041) reduction in progression in OA-BMLs grade favouring APS [14].

### 3.8. Secondary Outcomes

#### 3.8.1. Knee Pain

All studies assessed pain using PROMs such as visual analogue scales (VAS) [14,17,25,26,29], KOOS [13,14,15,29], or WOMAC [7,14,17,26].

A significant improvement in WOMAC pain subscale scores (*p* = 0.02) was observed in the study evaluating APS, compared to placebo, over 12 months of follow-up [14]. Similarly, the study comparing TissueGene-C with placebo reported a significant improvement in pain in the TissueGene-C group compared to placebo at 52 weeks follow-up [27,29]. Another study evaluating high-dose TissueGene-C with low-dose TissueGene-C found a significant improvement in pain within both groups (*p* < 0.001); the between-group difference, however, was not significant (*p* > 0.05) [17,28].

Two studies evaluating corticosteroids reported no significant benefit compared to placebo in pain reduction at 14 and 26 weeks of follow-up [13], or at 24 months [26]. Similarly, studies evaluating PRP [15] and sprifermin [7,23,25] reported no benefit, compared to placebo, in pain reduction over a follow-up period of 12 and 24 months. However, Lohmander et al. reported statistically significantly lower improvement in pain compared to placebo at 12 months follow-up (*p* = 0.0013) (Table 3) [24,25].

#### 3.8.2. Health-Related Quality-of-Life

Three studies reported HRQoL using a 36-Item Short Form Health Survey (SF-36) [14,26,27,29], and one study used the Assessment of Quality of Life–8 Dimension (AQoL-8D) tool [15]. None of the studies found a significant improvement in HRQoL assessed at 12 months or 24 months (Table 4) [14,15,26,27,29].

#### 3.8.3. Safety Outcomes

The overall safety profile of intra-articular therapies was acceptable, with no noticeable concerns (Table 5). No serious adverse events (SAEs) were reported with PRP, although the PRP group experienced more commonly encountered adverse events (AEs), such as knee joint pain, swelling, and stiffness after injections compared to the placebo [15]. Kon et al. demonstrated a favourable safety profile of APS at 12 months follow-up with no significant difference in the frequency and severity of AEs between groups with SAEs unrelated to the treatment [14].

Two studies evaluating sprifermin reported an acceptable safety profile with no treatment-related SAEs or AEs reported in both trials [7,23,25]. The typical local treatment-emergent AEs were arthralgia, joint swelling, and injection-site pain [7,23,25]. Similarly, TissueGene-C trialled in two studies showed no noticeable safety concerns. One study reported no significant difference in AEs [17,28], and the second reported joint inflammation, arthralgia, and effusion to be commonly experienced AEs in the TissueGene-C arm [27,29]. Both studies reported no SAEs related to treatment [17,27,28,29]. Likewise, corticosteroids assessed in two studies were reported to have no noticeable safety concerns [13,26]. While one study reported no SAEs [13], another found no significant difference in SAEs in the two arms (*p* = 0.06) [26].

## 4. Discussion

To the best of our knowledge, this is the first study to systematically review the efficacy and safety of intra-articular therapies for the treatment of KOA with a primary focus on structural changes assessed using OA-BMLs and symptomatic improvement assessed using knee pain.

This systematic review found mixed evidence from the included primary studies. High-quality evidence from the RCTs demonstrated improvement in the whole knee MRI-assessed OA-BMLs with high dose sprifermin at 6 to 12 months [25] and in PFJ OA-BMLs up to 12 months [7]. A significant reduction in OA-BML grade was seen with APS at 12 months of follow-up [14], and OA-BMLs improved in the high-dose cohort with TissueGene-C over the same follow-up period [17]. Similarly, a statistically significant reduction in OA-BML volume in the short term (14 weeks) was observed with intra-articular corticosteroids [13]. On the other hand, the beneficial effect of sprifermin on OA-BMLs was not significant when assessed from baseline to 12 months [25] and when accounted for the entire knee region at a longer follow-up (24 months) [7]. Another study assessing TissueGene-C found no differences in the progression of OA-BMLs when compared with placebo at 12 months [29]. The study that reported a positive effect of corticosteroids at 14 weeks found the difference in OA-BMLs levelled out at 26 weeks follow-up [13], whereas another study found no benefit of triamcinolone compared to placebo over 24 months [26]. The only study assessing PRP found no significant difference in OA-BML progression at 12 months of follow-up [15]. The improvement in knee pain outcome was reported in two studies evaluating TissueGene-C [17,29] and one study assessing APS. The remaining studies found no improvement in knee pain. The HRQoL outcomes assessed using the SF-36 and AQoL-8D in four studies found no significant improvement in scores with any of the intra-articular therapies compared with placebo [14,15,26,29].

Previous studies have found a discrepancy in the association between structural changes and pain in patients with OA [9]. However, OA-BMLs correlate with pain and changes in pain [30] in patients with KOA [9,11]. Furthermore, OA-BMLs are thought to drive OA-associated pain and may help predict treatment outcomes and prognosis [9,31]. Hence, researchers have argued that OA-BMLs could be the appropriate target for novel interventions that might reduce symptoms and improve the structural progression of KOA [9,31].

Two studies that evaluated sprifermin reported positive findings for improvements in OA-BMLs when considered for PFJ [7] or at a shorter follow-up [25]. Morphologically, positive findings in the PFJ can be attributed to its indirect effect on cartilage thickness [23] and the different loading patterns compared to the more load-bearing tibiofemoral joint (TFJ). Furthermore, less worsening of cartilage surface morphology in the PFJ may lead to an improvement in OA-BMLs at the PFJ [7]. However, an improvement in the whole knee region from 6 to 12 months and not from baseline to 12 months is not easily explained, and the exact causes are still to be understood [25]. The study evaluating APS showed the beneficial effect of APS, improving OA-BMLs with a significant improvement in knee pain, compared with placebo at 12 months [14]. However, the authors noted no improvement in cartilage and suggested future studies to confirm whether OA-BML improvements could be attributed to APS or if the observed improvements were the result of other unexplained factors [14].

TissueGene-C was evaluated in two studies that reported mixed findings. Cho et al.’s. study was constrained by a smaller sample size (n = 27), shorter follow-up duration, and no placebo control [17]. Guermazi et al. found no difference in OA-BMLs with TissueGene-C when compared to placebo, however an improvement in KOA structural features and other MRI markers such as Hoffa-synovitis and effusion-synovitis was observed [29]. Furthermore, the study found no improvement in meniscal damage or hypertrophic osteophyte formation [29]. Future studies of TissueGene-C in KOA should use a sufficient sample size and use a placebo-controlled study design.

The two studies evaluating corticosteroids uniformly demonstrated no benefit in OA-BML reduction in KOA [13,26] and found no association between OA-BMLs and knee pain [13]. Although Nielsen et al. found a significant positive effect at 14 weeks of follow-up, the effect levelled out at 26 weeks. The study concluded that there was no relation between corticosteroids and OA-BML volume [13]. This finding was consistent with the McAlindon et al. study that reported significantly greater cartilage volume loss and no improvements in OA-BMLs with triamcinolone compared with saline placebo. To be noted, earlier KOA trials have reported a strong placebo response to intra-articular injection, and a higher placebo effect has been a known phenomenon in OA studies [13,32]. Bennell et al. found no benefit of PRP compared to placebo for improvements in OA-BMLs, cartilage volume loss, or pain reduction and did not support the use of PRP for treating KOA [15]. The findings were inconsistent with earlier studies reporting the beneficial effects of PRP in pain reduction [33]. This inconsistency could be attributed to discrepancies in preparing PRP, injection regimens, outcome measures, and patient characteristics. Furthermore, the lack of blinding in the earlier trials may have influenced the positive outcome with PRP [15,33]. 

Additionally, although intra-articular therapies involving surgery were out of the scope of this paper, we note that studies using stem cell therapy, such as bone marrow aspirate concentrate (BMAC) or mesenchymal stem cells (MSCs), showed some promise by demonstrating regression of subchondral OA-BMLs, improvement in pain and subsequent reduction in progression to TKR [12,16]. However, further studies are warranted in this area as well. Additionally, given that poor knee alignment and resultant dynamic load are known to impact significantly on the natural history of KOA, this may contribute to the heterogenous OA-BML outcomes seen in our study [9,14]. Furthermore, variability among studies was observed in terms of MRI scoring tools used to assess OA-BMLs. While the majority of studies used tools such as WORMS and MOAKS, other studies, such as Nielsen et al., used computer-assisted segmentation (CAS) for OA-BML scoring, reflecting the heterogeneity of BML measurement [13]. Future studies should use validated tools (WORMS and MOAKS) to standardise the reporting of structural changes in KOA (OA-BMLs) to allow effective comparison across studies [34,35].

The safety of intra-articular therapies across the included studies was favourable, with no notable AEs or SAEs reported. The implication of these findings is of particular interest as it confirms the absence of any harmful effect of intra-articular therapies delivered in a clinical setting. Given that the intra-articular therapies in this review are safe in a controlled environment, we suggest further studies investigate their use in the clinic over a more extended period.

Notable strengths of this systematic review include a registered protocol-based method, exhaustive database and hand-searching, and a transparent risk of bias assessment. However, this study has certain limitations. Of note, we included only the RCTs that assessed intra-articular therapy delivered in a clinic setting through a non-operative procedure for treating KOA and reported OA-BMLs as an outcome. Considerable variability existed in reporting the OA-BML outcomes across the studies, limiting the possibility of a meta-analysis. Additional work could be done to assess the effect of intra-articular therapies on structural changes associated with pain in KOA. Future work should aim to incorporate outcomes such as osteophytes, effusion, synovitis, cartilage thickness, cartilage defect, and meniscal damage. Nonetheless, our study sets the priority for future systematic reviews to focus on structural outcomes and their association with symptom improvement in patients with KOA in this rapidly evolving research area.

## 5. Conclusions

This systematic review found mixed evidence on the efficacy of intra-articular therapies for improving OA-BMLs in patients with KOA. While high-dose sprifermin, TissueGene-C, and APS showed some promise for improving OA-BMLs, corticosteroids and PRP showed no improvement in OA-BMLs. In addition, no intra-articular therapy, except TissueGene-C and APS, showed any improvements in knee pain. Overall, although some of the studies were promising, the data is heterogenous, and more research is needed over a longer follow-up to support the use of intra-articular therapies for improvement in OA-BMLs in KOA.

## Figures and Tables

**Table 1:** Study design characteristics.

Study Author, Year, Country, Study Name (NCT ID)	Study Design	Study Duration & Follow-Up Period	Population	OA Type; Diagnosis	Age (Years), Mean (SD)	Patients (N) I/C	Gender, n (%)	BMI, Mean (SD)	Intervention Description	Control Description	Key Outcomes	Funding
Kon 2018; Italy; PROGRESS II (NCT02138890)	RCT, double-blinded, saline controlled, multi-centre	12 months	Patients with unilateral KOA	KOA; Radiographic KL grade 2–3	Intervention, y (range): 57 (41–68) Control, y (range): 54 (44–67)	N = 43 29/14	Intervention: Male/Female: 18/13 Control: Male/Female: 9/6	NR	Single-injection of APS	Saline injection (0.9% sodium chloride solution)	Change in OA-BML size was assessed using MRI MOAKS and radiographPRO assessed at 2 weeks and at 1-, 3-, 6-, and 12-months using VAS, WOMAC, KOOS, SF-36, CGI-S/C, PGI-S/C, and OMERACT-OARSI responder rate	Zimmer Biomet
Cho 2016; South Korea; NCT02341378	Prospective, randomized, single-blind trial	12 months	Patients who had OA of the knee, unresponsive to medical or physical therapy who have major lesions (less than 6 cm^2^) that were concentrated in one section of the knee and thought to be the primary cause of clinical symptoms	Evidence of grade 4 KOA as per ICRS criteria	Tissue Gene-C Low-dose group (group 1), Mean (range): 60 (46–72) Tissue Gene-C High-dose group (group 2), Mean (range): 58 (49–72)	N = 27 TissueGene-C Low-dose group (group 1) n: 14 Tissue Gene-C High-dose group (group 2) n: 13	Male: 6 Female: 21	NR	Low dose group: TissueGene-C [a 3:1 mixture of non-transduced chondrocytes and genetically engineered chondrocytes], at doses of 6 × 10^6^ cells	High dose group: TissueGene-C at doses 1.8 × 10^7^ cells	Modified version of WORMS used to assess changes in BME lesions, cartilage defect depth and surface area, articular bone surface and osteophytes, meniscus structure and signal, joint fluid, periarticular inflammation, and synovial inflammation	Kolon Life Science
Roemer 2016, Multinational; NCT01033994	Randomized, double blind, placebo-controlled trial	12 months	Patients were aged ≥40 years, had an established diagnosis of primary tibio-femoral KOA in the target knee	Primary tibio-femoral KOA according to ACR clinical and radiologic criteria with KL grade 2–3	100 μg group: 61.2 (9.1) Placebo group: 60.9 (6.9)	N = 75 57/18	100 μg group: Female 39 (68.4%) Placebo group: Female 12 (66.7%)	100 μg group: 30.5 (5.0) Placebo group: 31.5 (5.3)	Sprifermin treatment with 100 μg of dose	Matched placebo groups	OA-BMLs assessed using qMRI using modified WORMSChange in tibio-femoral compartment cartilage thickness assessed using qMRI	EMD Serono
McAlindon 2017; US; NCT01230424	Randomized, placebo-controlled, double-blind study	24 months	Patients aged 45 years or older with KOA and ultrasonographic evidence of effusion synovitis	Presence of KOA defined by the ACR criteria and KL grade 2–3	Overall: 58 (8) Triamcinolone: 59.1 (8.3) Saline: 57.2 (7.6)	N = 140 70/70	Female: 75 (54%) Triamcinolone: 37 (52.9%) Saline: 38 (54.3%)	Triamcinolone: 30.8 (5.1) Saline: 31.7 (6.6)	1 mL of triamcinolone (purchased from Bristol-Myers Squibb), 40 mg/mL, for injection administered every 12 weeks for 2 years	The comparator (saline) was 1 mL of 0.9% sodium chloride for injection (Hosperia Inc.) administered every 12 weeks for 2 years.	OA-BML volume assessed using semi-automated sagittal proton density fat–suppressed MRI PRO included WOMAC and SF-36Co-primary outcomes changed in knee cartilage volume in the index compartment, assessed using cartilage thickness	NIAMS and National Center for Advancing Translational Sciences, National Institutes of Health
Roemer 2020; Multinational; FORWARD (NCT01919164)	Randomized, Double Blind, Placebo-controlled, Multicenter	24 months	Patients aged 40–85 years with symptomatic radiographic primary femorotibial OA with medial minimum joint space width ≥2.5 mm in the target knee	Symptomatic radiographic KOA according to ACR criteria, KL grade 2–3	Placebo, Median age, years (range): 64.5 (41–83) Sprifermin 30 μg q12mo (N = 110), Median age, years (range): 66.5 (41–80) Sprifermin 60 μg q6mo (N = 111), Median age, years (range): 65.0 (41–80) Sprifermin 100 μg q12mo (N = 110), Median age, years (range): 65.0 (40–80) Sprifermin 100 μg q6mo (N = 110), Median age, years (range): 66.0 (44–84)	N = 549 441/108	C, female (%): 70.4% Sprifermin 30 μg q12mo (N = 110), female (%): 66.4% Sprifermin 60 μg q6mo (N = 111), female (%): 72.1% Sprifermin 100 μg q12mo (N = 110), female (%): 70% Sprifermin 100 μg q6mo (N = 110), female (%): 66.4%	Placebo, Median BMI, kg/m2 (range): 29.2 (19.5–46.3) Sprifermin 30 μg q12mo (N = 110), Median BMI, kg/m2 (range): 28.8 (18.6–51.3) Sprifermin 60 μg q6mo (N = 111), Median BMI, kg/m2 (range): 28.2 (18.6–44.5) Sprifermin 100 μg q12mo (N = 110), Median BMI, kg/m2 (range): 27.9 (17.5–43.5) Sprifermin 100 μg q6mo (N = 110), Median BMI, kg/m^2^ (range): 29.4 (21.2–43.3)	sprifermin (30 g or 100 g) administered as three weekly intra-articular injections in 6- or 12-month cycles	Placebo	Change in OA-BML assessed with qMRI using modified WORMS Changes in TFTJ cartilage thickness assessed using qMRI	Merck
Guermazi et al. 2017; US; NCT01221441	Multi-centre double-blind placebo-controlled phase II randomized clinical trial	1.5 years; 1 year	Patients 18–70 years of age with radiographic KOA and BMI between 18.5 to 45.5	KL grade 3 radiographic KOA as determined by the criteria of Kellgren and Lawrence	Tissue Gene-C: 55.9 (7.9) yearsPlacebo: 56.6 (9.4) years	N = 86 57/29	Female n (%): Tissue Gene-C: 37 (64.9%) Placebo: 17 (58.6)	NR	TissueGene-C	Saline placebo	OA-BML grade assessed using WORMSOther outcomes: meniscal damage, effusion-synovitis, and osteophytes assessed using WORMS	Kolon TissueGene
Bennell, 2021; Australia; RESTORE (ACTRN12617000853347)	RCT	24 months; 12 months	Community-based participants aged 50 years or older with symptomatic mild to moderate medial KOA	Symptomatic medial KOA with KL grade 2–3	PRP: 62.2 (6.3) years Placebo: 61.6 (6.6) years	N = 288 144/144	Female n (%): PRP: 85 (59.0) Placebo: 84 (58.3)	PRP: 29.0 (3.7) Placebo: 29.6 (4.5)	3 intra-articular PRP injections at weekly intervals	Saline placebo	MRI assessed medial distal femur and proximal tibia OA-BML sizeMRI-measured medial tibial cartilage volumePRO knee pain severity assessed using KOOS, quality-of-life assessed with AQoL-8D	NHMRC Regen Lab SA provided the commercial kits free of charge
Nielsen 2018; Denmark; EudraCT 2012-002607-18	Randomised placebo controlled, outcome assessor blinded trial	26 weeks; 14 weeks	Participant from OA outpatient clinic aged 40 or older and BMI of 35 or lesser.	Tibiofemoral OA according to the ACR-criteria	Corticosteroid: 62.1 (9.4) Placebo: 65.4 (8.3)	N = 86 41/45	Female n (%) Corticosteroid: 22 (53.7%) Placebo: 30 (66.7%)	Corticosteroid: 29.2 (4.1) Placebo: 29.0 (3.4)	Corticosteroid: intra-articular 1-mL injection of methyl prednisolone acetate (Depo-Medrol), 40 mg/mL, dissolved in 4 mL of lidocaine hydrochloride (10 mg/mL)	Placebo: a 1-mL isotonic saline injection mixed with 4 mL of lidocaine hydrochloride (10 mg/mL)	MRI assessed change in OA-BML volumePRO pain assessed using KOOS	Danish Council for Independent Research, Medical Science and by the Oak Foundation, Association of Danish Physiotherapists, Lundbeck Foundation, and Capital Region of Denmark

ACR: American College of Rheumatology classification criteria; APS: autologous protein solution; AQoL-8D: Assessment of Quality of Life–8 Dimension score; BME: Bone marrow edema; CGI-S: Clinical Global Impression of Severity; CGI-S/C: Clinical Global Impression of Severity/Change; cMFTC: Central Medial Femorotibial Compartment; KL grade: Kellgren-Lawrence grading scale; C: Control arm; I: Intervention arm; ICRS criteria: International Cartilage Repair Society; KOA: knee osteoarthritis; KOOS: Knee Injury, and Osteoarthritis Outcome Score; LR-PRP: Leukocyte Rich-Platelet-rich Plasma; MOAKS: MRI Osteoarthritis Knee Score; NIAMS: National Institute for Arthritis and Musculoskeletal Disorders and Skin Diseases; NR: not reported; OA-BML: bone marrow lesion; OMERACT-OARSI: Outcome Measures in Rheumatology–Osteoarthritis Research Society International; PGI-S/C, Patient Global Impression of Severity/Change; PRO: Patient Reported Outcomes; PRP: Platelet-rich Plasma; RCT: Randomized Controlled Trial; SF-36: 36-Item Short Form Health Survey; VAS: Visual Analogue Scale; WOMAC: Western Ontario and McMaster Universities Osteoarthritis Index; WORMS: Whole Organ Magnetic Resonance Imaging Score.

**Table 2:** Changes in MRI-measured OA-BMLs after treatment at longest reported follow-up.

Study	Outcome Details	Intervention/Control	Longest Follow-Up	Baseline (Intervention)	Baseline (Control)	*p*-Value	Follow-Up (Intervention)	Follow-Up (Control)	*p*-Value	Conclusion
**OA-BMLs assessed using modified WORMS**										
Cho 2016 NCT02341378	Mean OA-BML score (% of maximum possible score)	High-dose TissueGene-C/Low-dose TissueGene-C	12 months	10 (31%)	7 (22%)	NA	8 (25%)	7 (22%)	NA	In high dose group BME lesions improved from pre-treatment at 12 months.
Roemer 2016 NCT01033994	Grades of OA-BML * (95% CI)	Sprifermin 100 μg cohort/Placebo	12 months	3.3 (2.5, 4.1)	3.3 (1.4, 5.2)	0.642	Change from baseline −0.20 (−0.67, 0.28)	Change from baseline 0.22 (−0.62, 1.07)	0.237	Significant improvement in OA-BMLs from 6 to 12 months; no significant improvement from baseline to 12 months.
Roemer 2020 NCT01919164	Mean OA-BML score * (95% CI)	Sprifermin 100 mg q12mo/Placebo	24 months	4.0 (3.4, 4.6)	4.1 (3.5, 4.8)		Change from baseline 0.1 (−0.3, 0.5)	Change from baseline 0.1 (−0.4, 0.5)	NA	Positive effects associated with Sprifermin were observed for cartilage morphology changes at PFJ. However, no difference was observed between treatment groups in OA-BML changes across entire knee.
Guermazi 2017 NCT01221441	Progression in OA-BML grade	TissueGene-C/Placebo	12 months	NA	NA		Any OA-BML progression 66.2%	Any OA-BML progression 60.6%	0.612	No differences were observed with regard to the progression of OA-BMLs.
**OA-BMLs assessed using MRI (varied methods)**										
McAlindon 2017 NCT01230424	OA-BML volume (log) ^§^ assessed using validated sqMRI approach ^†^	Triamcinolone/Saline	24 months	7.79 (6.47, 9.11)	6.80 (5.47, 8.13)	NA	Change from baseline 0.89 (−0.29, 2.08)	Change from baseline 1.11 (−0.33, 2.57)	0.80	No significant improvement was observed in OA-BML between triamcinolone and saline group.
Bennell 2021 ACTRN12617000853347	Progression in OA-BML grade reported as n (%) ^¶^	PRP/Placebo	12 months	NA	NA	NA	34 (24.3%)	25 (18.9%)	0.31	No significant change in OA-BMLs was observed at 12 month follow-up.
Nielsen 2018 EudraCT 2012-002607-18 NCT01945749	Mean (95% CI) difference in relative OA-BML volume ^||^*	Corticosteroid/Placebo	14 weeks	12.0 (7.8, 16.3)	12.5 (8.2, 16.8)	NA	Change from baseline −1.1 (−3.5, 1.3)	Change from baseline 2.7 (0.4, 4.9)	0.03	A significant difference was observed in OA-BML volume at 14-week follow-up; however, difference in OA-BML volume levelled out at the 26 week follow-up and no association was found between KOOS and OA-BML volume.
			26 weeks				0.8 (−1.7, 3.3)	1.6 (−1.0, 4.1)	0.65	
**OA-BMLs assessed using MOAKS**										
Kon 2018 NCT02138890	Change from baseline in OA-BML grade reported as number of patients for each category ^‡^	APS/Placebo	12 months	NA	NA	NA	Change from baseline in OA-BML grade, n −1 change: 2 No change: 26 +1 change: 2	Change in OA-BML from baseline, n No change: 10 +1 change: 3 + 2 change: 1	0.041	A significant difference between group was found in change from baseline to 12 months in OA-BML size in favour of APS.

* Delta sum approach: Adds the absolute scores of all sub-regions combined per compartment or for the whole knee. ^§^ Higher natural log values for OA-BMLs denote greater volumes affected by these findings. ^†^ Zhang *Biomed Res Int.* 2015; 2015:634275. ^¶^ OA-BMLs graded in the medial distal femur and medial proximal tibia as 0 to 3 (0, absent; 1, occupies less than one-third of the region; 2, occupies one-third to two-thirds of the region; and 3, occupies greater than two-thirds of the region). Progression was defined as an increase in OA-BML grade of ≥1 in either the medial distal femur or medial proximal tibia between baseline and 12 months. || Nielsen *BMC Musculoskelet Disord*. 2014; 15:447. ^‡^ The format of reported data is the number of patients with –3; –2; –1; No change; +1; +2; +3 OA-BML grade change. BME: Bone marrow edema; MOAKS: MRI Osteoarthritis Knee Score; NA: not applicable/available; PFJ: patellofemoral joint; q12mo: every 12 months active cycle; sqMRI: semi-quantitative MRI; WORMS: Whole Organ Magnetic Resonance Imaging Score.

**Table 3:** Changes in knee pain after treatment at longest reported follow-up.

Study	Outcome Details	Intervention/Control	Longest Follow-Up	Baseline (Intervention)	Baseline (Control)	*p*-Value	Follow-Up (Intervention)	Follow-Up (Control)	*p*-Value	Conclusion
Cho 2016 * **NCT02341378**	VAS, mean	High-dose TissueGene-C/Low-dose TissueGene-C	24 weeks	48	52	0.52	Change from baseline −20	Change from baseline −24	0.48	No significant between group difference in pain reduction was observed. However, both the group showed significant improvement from baseline when assessed within group.
	WOMAC pain, mean			6	6	0.98	Change from baseline −3	Change from baseline −3	0.58	
Roemer 2020 ** **NCT01919164**	WOMAC, mean	Sprifermin 100 mg q12mo/Placebo	24 months	NA	NA	NA	Change from baseline −21	Change from baseline −22	NS	No significant differences were observed in mean absolute change from baseline for WOMAC pain.
Guermazi 2017 *** **NCT01221441**	VAS, LS mean	TissueGene-C/Placebo	12 months	NA	NA	NA	−34.9	−24.8	0.03	A significant improvement in pain was observed at 52 weeks in TissueGene-C group compared to placebo.
	KOOS, mean (SD)			46.9 (15.7)	44.8 (14.5)	NA	Change from baseline 26.9 (21.3)	Change from baseline 15.1 (26.3)		
**Kon 2018** **NCT02138890**	VAS	APS/Placebo	12 months	5.5	6.5	NS	% improvement from baseline 49%	% improvement from baseline 13%	0.06	A significant improvement was observed in WOMAC pain score in patients receiving APS compared with placebo.
	WOMAC pain			11.4	11.8	NS	65%	41%	0.02	
	KOOS pain			39.9	37.9	NS	NR	NR	NS	
**McAlindon 2017** **NCT01230424**	VAS, mean (95% CI)	Triamcinolone/Saline	24 months	30.8 (22.9, 38.7)	35.4 (27.6, 43.2)	NA	Change from baseline −2.7 (−11.9, 6.6)	Change from baseline −7.6 (−15.4, 0.16)	0.26	Triamcinolone compared to saline placebo showed no significant difference in knee pain over 24 months follow-up.
	WOMAC pain, mean (95% CI)			7.50 (6.3, 8.6)	8.2 (7.0, 9.3)	NA	−1.2 (−1.9, −0.58)	−1.9 (−2.52, −1.23)	0.17	
**Bennell 2021** **ACTRN12617000853347**	KOOS pain, mean (SD)	PRP/Placebo	12 months	52.9 (15.2)	53.5 (13.5)		Change from baseline 15.1 (18.9)	Change from baseline 11.9 (17.6)	0.12	PRP compared with placebo did not result in a significant difference in pain reduction over 12 months follow-up.
**Nielsen 2018** **EudraCT 2012-002607-18** **NCT01945749**	KOOS pain, mean (95% CI)	Corticosteroid/Placebo	14 weeks	52.6 (48.8, 56.3)	55.9 (51.3, 60.5)	NA	Change from baseline 14.3 (10.2, 18.3)	Change from baseline 14.6 (10.7, 18.4)	0.92	No symptomatic difference was found between the intervention and placebo group. Furthermore, no association between change in OA-BMLs and knee pain.
			26 weeks				13.3 (8.6, 18.1)	16.7 (12.1, 21.2)	0.32	
Roemer 2016 **** **NCT01033994**	VAS	Sprifermin 100 μg cohort/Placebo	12 months	No increase in pain VAS observed in both the groups						A statistically significantly lower improvement in pain was observed with sprifermin compared to placebo at 12 months follow-up.
	WOMAC pain, mean (SD)			10.4 (2.8)	10.1 (2.6)	NA	−2.87 (4.76)	−5.56 (4.17)	0.001	

* Reported in Ha et al. *Hum Gene Ther Clin Dev.* 2015;26(2):125–130. ** Reported in Hochberg. *JAMA*. 2019;322(14):1360–1370. ^***^ Reported in Cherian et al. *Osteoarthritis Cartilage.* 2015;23(12):2109–2118. **** Reported in Lohmander et al. *Arthritis Rheumatol.* 2014;66(7):1820–1831. APS: autologous protein solution; KOOS: Knee Injury and Osteoarthritis Outcome Score; LS mean: least square mean; NA: not available; NS: not significant; OA-BML: Bone marrow lesion; PRP: platelet rich plasma; VAS, Visual Analog Scale; WOMAC, Western Ontario and McMaster Universities Osteoarthritis Index.

**Table 4:** Quality of life outcomes at longest reported follow-up.

Study	Outcome Details	Intervention/Control	Longest Follow-Up	Baseline (Intervention)	Baseline (Control)	*p*-Value	Follow-Up (Intervention)	Follow-Up (Control)	*p*-Value	Conclusion
**Kon 2018** **NCT02138890**	SF-36 score (Mental) mean (SD)	APS	12 months	51.5	50.8	NS	NA	NA	NA	There were no significant differences in the SF-36 outcome measures.
	SF-36 score (Physical) mean (SD)	Placebo		35.8	33.9		NA	NA	NA	
**McAlindon 2017** **NCT01230424**	SF-36 score (Mental) mean (SD)	Triamcinolone	24 months	36.7 (9.1)	35.4 (9.7)	NA	N	NA	NA	NA
	SF-36 score (Physical) mean (SD)	Saline		52.6 (10.2)	52.2 (10.0)	NA	NA	NA	NA	
**Bennell 2021** **ACTRN12617000853347**	AQoL8D, mean (SD)	PRP/Placebo	12 months	0.72 (0.15)	0.72 (0.16)		Change from baseline 0.04 (0.13)	Change from baseline 0.04 (0.12)	0.91	No significant change in HRQoL at 12 month follow-up.
Guermazi 2017 * **NCT01221441**	Overall SF-36, LS mean (95% CI)	TissueGene-C/Placebo	12 months	NA	NA	NA	Difference between treatment and placebo −0.4 (−5.4, 4.6)		0.88	No significant difference in SF-36 assessed overall HRQoL at 52 weeks.

* Reported in Cherian et al. *Osteoarthritis Cartilage.* 2015;23(12):2109–2118. AQoL-8D: Assessment of Quality of Life–8 Dimension score PRO tool; HRQoL: health-related quality of life; LS mean: least square mean; NA: not available; SD: standard deviation; SF-36: 36-Item Short Form Health Survey.

**Table 5:** Safety Outcomes.

Study	Intervention/Control	Safety Outcomes			
		AEs	SAEs	Additional Details	Conclusion
**Kon 2020** **NCT02138890**	APS, n (%)	14 (45.2%)	2 (bladder cancer and kidney stone)	Total number of AEs: 48	APS displayed a positive safety profile; no significant differences in frequency and severity of AEs between groups. SAEs were unrelated to treatment.
	Placebo, n (%)	6 (40.0%)	1 (meniscus tear)	Total number of AEs: 17	
Cho 2016 * **NCT02341378**	High-dose TissueGene-C, n (%)	Major AEs 10 (71)	None	High-dose treatment group had a higher incidence of AEs, which may be attributed to a dose-dependent increase in TGF-b.	No significant difference for either AEs or ADRs and are unlikely to be clinically relevant. Moreover, there were no serious AEs noted.
	Low-dose TissueGene-C, n (%)	Major AEs 8 (57)	None		
Roemer 2016 ** **NCT01033994**	Sprifermin 100 μg, n (%)	4 (66.7%)	17 (27)	Higher percentages of patients receiving Sprifermin as compared to placebo experienced one or more treatment emergent AEs.	No significant difference in treatment-emergent AEs, SAEs, or acute inflammatory reactions between the combined Sprifermin group and the placebo group.
	Placebo, n (%)	3 (50)	7 (16)		
**McAlindon 2017** **NCT01230424**	Triamcinolone, n	52	No significant differences in SAEs (*p* = 0.06)	Treatment-related AEs: 5 (1 facial flushing, 4 injection site pain) SAEs: Worsening hypertension: 1	Significantly more AEs were reported in the saline group compared to the triamcinolone group.
	Saline, n	63		Treatment-related AEs: 3 (1 cellulitis, 2 injection site pain) SAEs: Worsening hypertension: 2	
Romer 2020 *** **NCT01919164**	Sprifermin 100 μg, n (%)	99 (92.5%)	17 (15.3%)	Local treatment-emergent AEs were similar across treatment groups and most commonly consisted of arthralgia, joint swelling, and injection-site pain.	Treatment-emergent AEs were mostly mild/moderately severe and not related to treatment. SAEs were not considered related to treatment.
	Placebo, n (%)	101 (91%)	27 (25.2%)		
Guermazi 2017 **** **NCT01221441**	TissueGene-C, n (%)	58 (87%)	2	45 (67%) patients experienced AEs related to the study drug.	AEs related to treatment (TissueGene-C) were joint inflammation (patients, n = 19), arthralgia (14), and effusion (14). SAEs were not related to treatment.
	Placebo, n (%)	27 (77%)	1		
**Bennell, 2021** **ACTRN12617000853347**	PRP, n (%)	90	None	Knee joint pain: 25 (18.1%); Knee swelling: 3 (2.2%); Knee stiffness: 5 (3.6%); Other lower limb musculoskeletal symptoms: 31 (22.5%); Upper body musculoskeletal symptoms: 13 (9.4%); Medical condition (non-musculoskeletal): 13 (9.4%)	There were no SAEs observed. AEs were minor and transient. More participants reported knee joint pain, swelling, and stiffness after injections in the PRP group compared to placebo.
	Placebo, n (%)	78	None	Knee joint pain: 21 (15.0%); Knee swelling: 0; Knee stiffness: 0; Other lower limb musculoskeletal symptoms: 23 (16.4%); Upper body musculoskeletal symptoms: 18 (12.9%); Medical condition (non-musculoskeletal): 16 (11.4%)	
**Nielsen 2018** **EudraCT 2012-002607-18**	Corticosteroid, n	1	None	NA	No SAE reported in any arm. No noticeable safety concerns raised in this study.
	Placebo, n	3	None	NA	

* Reported in Ha et al. *Hum Gene Ther Clin Dev.* 2015;26(2):125–30. ** Reported in Lohmander et al. *Arthritis Rheumatol.* 2014;66(7):1820–31. *** Reported in Hochberg. *JAMA.* 2019;322(14):1360–1370. **** Reported in Cherian et al. *Osteoarthritis Cartilage.* 2015;23(12):2109–2118. ADRs: adverse drug reactions; AEs: adverse events; APS: autologous protein solution; PRP: platelet rich plasma; SAEs: serious adverse events; TGF-β1: transforming growth factor; NA: not applicable; NR: not reported.

## Data Availability

Not applicable.

## Supplementary Materials

The following supporting information can be downloaded at: https://www.mdpi.com/article/10.3390/ph15121555/s1, Supplementary Figure S1: PRISMA 2020 flow diagram. Table S1: Ovid Medline search strategy. Table S2: Search terms used for Clinicaltrial.gov and ANZCTR. Table S3: List of ongoing trials of intra-articular injectables for the treatment of knee OA. Table S4: OMERACT-OARSI response. Table S5: PRISMA 2020 checklist.
