# Supplementary material for: How Effective Are Non-Operative Intra-Articular Treatments for Bone Marrow Lesions in Knee Osteoarthritis in Adults? A Systematic Review of Controlled Clinical Trials
Source: Pharmaceuticals (Basel). 2022 Dec 14;15(12):1555. doi: 10.3390/ph15121555 (PMC9787030; doi:10.3390/ph15121555)
Supplement: Supplementary file 1 [file pharmaceuticals-15-01555-s001.zip › pharmaceuticals-2077562-supplementary.pdf]

Supplement Figure S1: PRISMA 2020 flow diagram

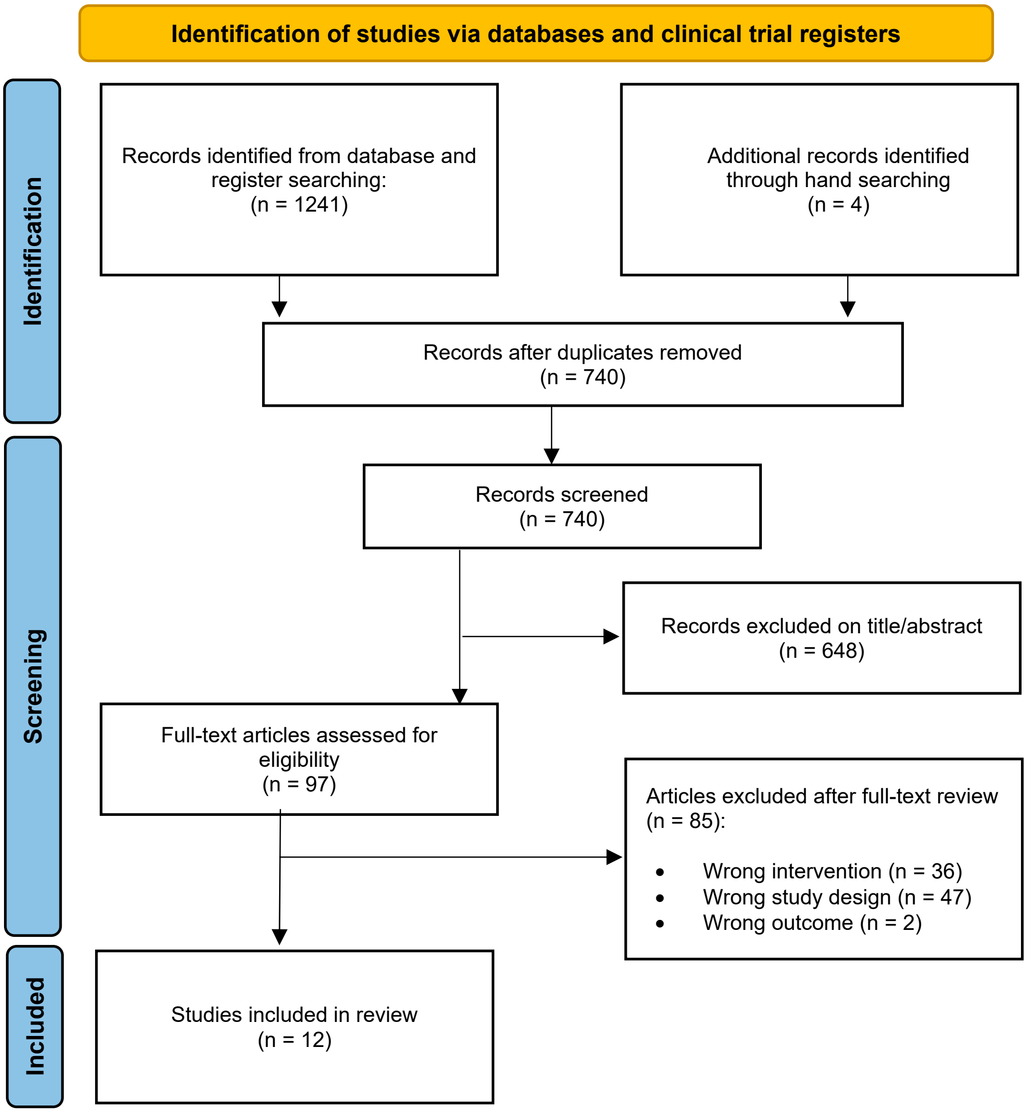

From: Page MJ, McKenzie JE, Bossuyt PM, Boutron I, Hoffmann TC, Mulrow CD, et al. The PRISMA 2020 statement: an updated guideline for reporting systematic reviews. *Syst Rev* 10, 89 (2021). https://doi.org/10.1186/s13643-021-01626-4

Supplement Table S1: Ovid Medline search strategy

1	Bone Marrow/ and (Edema/ or Contusions/)
2	(bone adj5 (bruis* or oedema* or edema* or contusion* or lesion*)).ti,ab,kf.
3	or/1-2
4	exp Osteoarthritis/
5	osteoarthrit*.ti,ab,kf.
6	arthritis/
7	arthriti*.ti,ab,kf.
8	(degenerat* adj3 joint*).ti,ab,kf.
9	arthrosis.ti,ab,kf.
10	Joint Diseases/
11	arthropathy.ti,ab,kf.
12	Arthralgia/
13	(joint adj3 (pain* or disease*)).ti,ab,kf.
14	arthralgia*.ti,ab,kf.
15	or/4-14
16	Knee/
17	exp Knee Joint/
18	knee*.ti,ab,kf.
19	or/16-18
20	randomized controlled trial.pt.
21	controlled clinical trial.pt.
22	(randomized or randomised).ti,ab.
23	placebo.ti,ab.
24	drug therapy.sh.
25	randomly.ti,ab.
26	trial.ti,ab.
27	groups.ti,ab.
28	or/20-27
29	exp animals/ not humans/
30	28 not 29
31	and/3,15,19,30

Supplement Table S2: Search terms used for Clinicaltrial.gov and ANZCTR

Trial Registry	Terms
Clinicaltrial.gov	"bone marrow oedema"<br>"bone marrow edema"<br>"bone marrow lesion" AND osteoarthritis<br>"bone marrow lesion" AND arthritis<br>"bone marrow lesion" AND "joint pain"<br>"bone marrow lesion" AND "degenerative joint"<br>"bone marrow lesion" AND arthralgia
ANZCTR	"bone marrow oedema"<br>"bone marrow lesion"<br>"bone marrow edema"

Supplement Table S3: List of ongoing trials of intra-articular injectables for the treatment of knee OA

S.No	Source	Registration Numbers	Title	Acronym	Status	Study Results	Conditions	Interventions	Outcome Measures	Age	Phases	Enrollment	Study Designs	Start Date	Country
1	Clinicaltrial.gov	NCT05081921	Clinical Trial to Evaluate Safety and Efficacy of MesoCellA-Ortho Tissue-Engineered Advanced Therapy Product in Patients With Osteoarthrosis and Civilisation Diseases (BioMiStem-CT)	BioMiStem-CT	Recruiting	No Results posted	OA knee	Drug: MesoCellA-Ortho administration |Drug: HA administration	Change in BML |Knee pain and function (KOOS and NRS)|SF-36 QoL|AEs | Change in T2 MRI	40 Years to 70 Years (Adult, Older Adult)	Phase 1-2	200	Allocation: Randomized|Intervention Model: Parallel Assignment|Masking: Quadruple (Participant, Care Provider, Investigator, Outcomes Assessor)|Primary Purpose: Treatment	05/01/2022	Poland
2	ANZCTR	ACTRN12620000870954	Evaluating the efficacy and cost-effectiveness of stem cell injections in people with mild to moderate knee osteoarthritis: a randomised placebo-controlled trial (The SCUlpTOR trial)	SCUlpTOR trial	Recruiting	No Results posted	OA knee	Drug: 2.5 x 10^7 cell culture-expanded mesenchymoangioblast-derived |Drug: Placebo	Change in BML |Knee pain and function (VAS and WOMAC)|KOOS QoL|AEs | Change in T2 MRI	eople >=40 years of age	Phase 3	440	Allocation: Randomized|Intervention Model: Parallel Assignment|Masking: Quadruple (Participant, Care Provider, Investigator, Outcomes Assessor)|Primary Purpose: Treatment	16/02/2022	Australia

OA: osteoarthritis; COL: colchicine; PLB: placebo; HA: Hyaluronic acid; MSCs: mesenchymal stem cells; KOOS: Knee injury and Osteoarthritis Outcome Score; NRS: Numerical Rating Scale

Searched from: Clinicaltrial.gov from https://clinicaltrials.gov/ on 13 Sep 2022; ANZCTR from https://www.anzctr.org.au/ on 13 Sep 2022;

Supplement Table S4: OMERACT-OARSI response

Study	Intervention/control	Follow-up	OMERACT-OARSI response		Conclusion
			Yes	No	
**Kon 2020 NCT02138890**	APS, n (%)	12 months	19 (65.5%)	10 (34.5%)	No significant difference between groups.
	Placebo, n (%)		7 (50%)	7 (50%)	
**Guermazi 2017* NCT01221441**	TissueGene-C	12 months	**Response rate** 58.3%		10 withdrawn patients had improvements in pain and/or function which correlated to a positive response as per modified OMERACT-OARSI criteria, whereas 16 patients did not.
	Placebo		42.9%		

* Reported in Cherian et al. *Osteoarthritis Cartilage.* 2015;23(12):2109-2118
APS: autologous protein solution; OMERACT-OARSI: Outcome Measures in Rheumatology–Osteoarthritis Research Society International

Supplement Table S5: PRISMA 2020 checklist

Section and Topic	Item #	Checklist item	Location where item is reported
**TITLE**			
Title	1	Identify the report as a systematic review.	1
**ABSTRACT**			
Abstract	2	See the PRISMA 2020 for Abstracts checklist.	1
**INTRODUCTION**			
Rationale	3	Describe the rationale for the review in the context of existing knowledge.	3-4
Objectives	4	Provide an explicit statement of the objective(s) or question(s) the review addresses.	4
**METHODS**			
Eligibility criteria	5	Specify the inclusion and exclusion criteria for the review and how studies were grouped for the syntheses.	4-5
Information sources	6	Specify all databases, registers, websites, organisations, reference lists and other sources searched or consulted to identify studies. Specify the date when each source was last searched or consulted.	4-5<br>Supplement<br>Protocol
Search strategy	7	Present the full search strategies for all databases, registers and websites, including any filters and limits used.	4-5<br>Supplement<br>Protocol
Selection process	8	Specify the methods used to decide whether a study met the inclusion criteria of the review, including how many reviewers screened each record and each report retrieved, whether they worked independently, and if applicable, details of automation tools used in the process.	4-5<br>Protocol
Data collection process	9	Specify the methods used to collect data from reports, including how many reviewers collected data from each report, whether they worked independently, any processes for obtaining or confirming data from study investigators, and if applicable, details of automation tools used in the process.	4-5<br>Protocol
Data items	10a	List and define all outcomes for which data were sought. Specify whether all results that were compatible with each outcome domain in each study were sought (e.g. for all measures, time points, analyses), and if not, the methods used to decide which results to collect.	4-5<br>Protocol
	10b	List and define all other variables for which data were sought (e.g. participant and intervention characteristics, funding sources). Describe any assumptions made about any missing or unclear information.	4-5<br>Protocol
Study risk of bias assessment	11	Specify the methods used to assess risk of bias in the included studies, including details of the tool(s) used, how many reviewers assessed each study and whether they worked independently, and if applicable, details of automation tools used in the process.	4-5<br>Protocol
Effect measures	12	Specify for each outcome the effect measure(s) (e.g. risk ratio, mean difference) used in the synthesis or presentation of results.	4-5<br>Protocol
Synthesis methods	13a	Describe the processes used to decide which studies were eligible for each synthesis (e.g. tabulating the study intervention characteristics and comparing against the planned groups for each synthesis (item #5)).	4-5<br>Protocol
	13b	Describe any methods required to prepare the data for presentation or synthesis, such as handling of missing summary statistics, or data conversions.	4-5<br>Protocol
	13c	Describe any methods used to tabulate or visually display results of individual studies and syntheses.	4-5

Section and Topic	Item #	Checklist item	Location where item is reported
			Protocol
	13d	Describe any methods used to synthesize results and provide a rationale for the choice(s). If meta-analysis was performed, describe the model(s), method(s) to identify the presence and extent of statistical heterogeneity, and software package(s) used.	4-5<br>Protocol
	13e	Describe any methods used to explore possible causes of heterogeneity among study results (e.g. subgroup analysis, meta-regression).	NA
	13f	Describe any sensitivity analyses conducted to assess robustness of the synthesized results.	NA
Reporting bias assessment	14	Describe any methods used to assess risk of bias due to missing results in a synthesis (arising from reporting biases).	NA
Certainty assessment	15	Describe any methods used to assess certainty (or confidence) in the body of evidence for an outcome.	NA
**RESULTS**			
Study selection	16a	Describe the results of the search and selection process, from the number of records identified in the search to the number of studies included in the review, ideally using a flow diagram.	6-9<br>Supplement
	16b	Cite studies that might appear to meet the inclusion criteria, but which were excluded, and explain why they were excluded.	6-9<br>Supplement
Study characteristics	17	Cite each included study and present its characteristics.	6-9<br>Supplement
Risk of bias in studies	18	Present assessments of risk of bias for each included study.	6-9<br>Supplement
Results of individual studies	19	For all outcomes, present, for each study: (a) summary statistics for each group (where appropriate) and (b) an effect estimate and its precision (e.g. confidence/credible interval), ideally using structured tables or plots.	6-9<br>Supplement
Results of syntheses	20a	For each synthesis, briefly summarise the characteristics and risk of bias among contributing studies.	6-9<br>Supplement
	20b	Present results of all statistical syntheses conducted. If meta-analysis was done, present for each the summary estimate and its precision (e.g. confidence/credible interval) and measures of statistical heterogeneity. If comparing groups, describe the direction of the effect.	6-9<br>Supplement
	20c	Present results of all investigations of possible causes of heterogeneity among study results.	NA
	20d	Present results of all sensitivity analyses conducted to assess the robustness of the synthesized results.	NA
Reporting biases	21	Present assessments of risk of bias due to missing results (arising from reporting biases) for each synthesis assessed.	NA
Certainty of evidence	22	Present assessments of certainty (or confidence) in the body of evidence for each outcome assessed.	NA
**DISCUSSION**			
Discussion	23a	Provide a general interpretation of the results in the context of other evidence.	10-12
	23b	Discuss any limitations of the evidence included in the review.	10-12
	23c	Discuss any limitations of the review processes used.	10-12
	23d	Discuss implications of the results for practice, policy, and future research.	10-12
**OTHER INFORMATION**			
Registration	24a	Provide registration information for the review, including register	2 and 4

Section and Topic	Item #	Checklist item	Location where item is reported
and protocol		name and registration number, or state that the review was not registered.	
	24b	Indicate where the review protocol can be accessed, or state that a protocol was not prepared.	4
	24c	Describe and explain any amendments to information provided at registration or in the protocol.	Protocol
Support	25	Describe sources of financial or non-financial support for the review, and the role of the funders or sponsors in the review.	12
Competing interests	26	Declare any competing interests of review authors.	12
Availability of data, code and other materials	27	Report which of the following are publicly available and where they can be found: template data collection forms; data extracted from included studies; data used for all analyses; analytic code; any other materials used in the review.	NA

From: Page MJ, McKenzie JE, Bossuyt PM, Boutron I, Hoffmann TC, Mulrow CD, et al. The PRISMA 2020 statement: an updated guideline for reporting systematic reviews. *Syst Rev* 10, 89 (2021). https://doi.org/10.1186/s13643-021-01626-4
